# Supplementary material for: Apoptotic and DNA Damage Effect of 1,2,3,4,6-Penta-O-galloyl-beta-D-glucose in Cisplatin-Resistant Non-Small Lung Cancer Cells via Phosphorylation of H2AX, CHK2 and p53
Source: Cells. 2022 Apr 14;11(8):1343. doi: 10.3390/cells11081343 (PMC9026497; doi:10.3390/cells11081343)
Supplement: Supplementary file 1 [file cells-11-01343-s001.zip › cells-1582698-supplementary.pdf]

cells

Supplementary Materials

Apoptotic and DNA Damage Effect of 1,2,3,4,6-Penta-O-galloyl-beta-D-glucose in Cisplatin-Resistant Non-Small Lung Cancer Cells via Phosphorylation of H_2AX, CHK2 and p53

Ji-Hyun Kim, Eunji Im, Jihyun Lee, Hyo-Jung Lee, Deok Yong Sim, Ji Eon Park, Chi-Hoon Ahn, Hyeon Hee Kwon, Bum Sang Shim, Bonglee Kim and Sung-Hoon Kim

Molecular Cancer Target Herbal Research Laboratory, College of Korean Medicine, Kyung Hee University, Seoul 02447, Korea; kimji77@pusan.ac.kr (J.-H.K.); ji4137@khu.ac.kr (E.I.); jhlee096@naver.com (J.L.); hyonice77@khu.ac.kr (H.-J.L.); simdy0821@khu.ac.kr (D.Y.S.); jieon77@khu.ac.kr (J.E.P.); ach2565@khu.ac.kr (C.-H.A.); dwfqwd223@hanmail.net (H.H.K.); eshimbs@khu.ac.kr (B.S.S.), bongleekim@khu.ac.kr (B.K.)

Correspondence: sungkim7@khu.ac.kr; Tel.: +82-2-961-9223

(A)

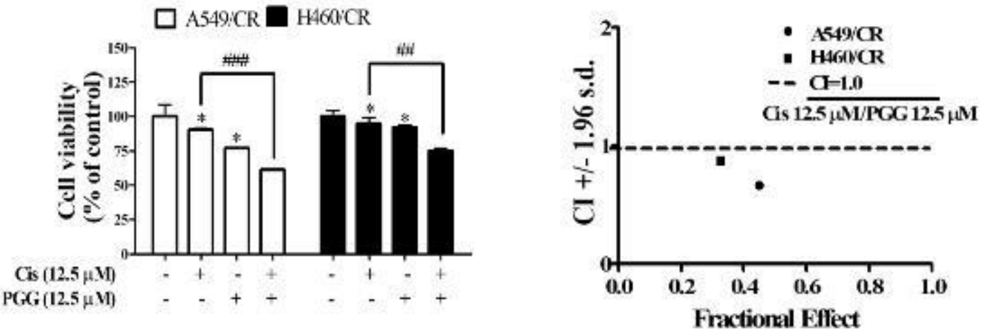

(B)

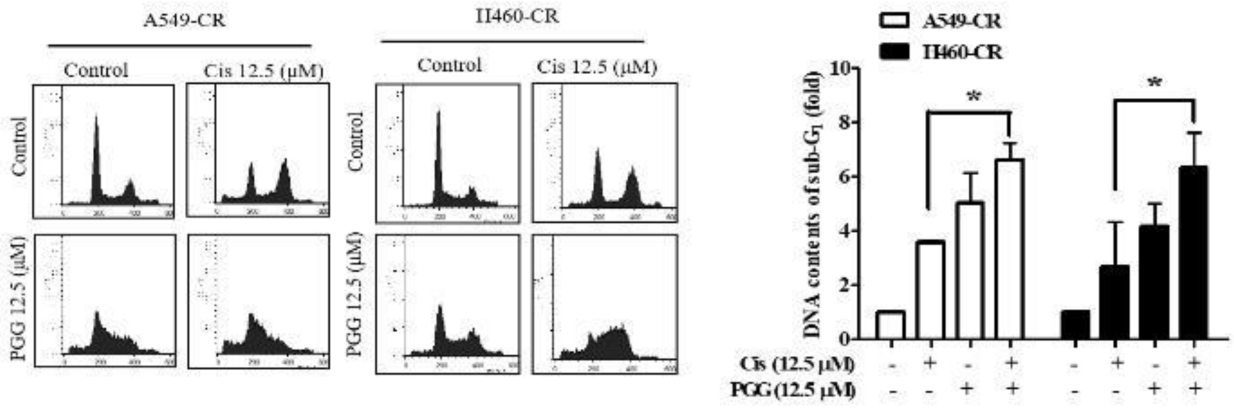

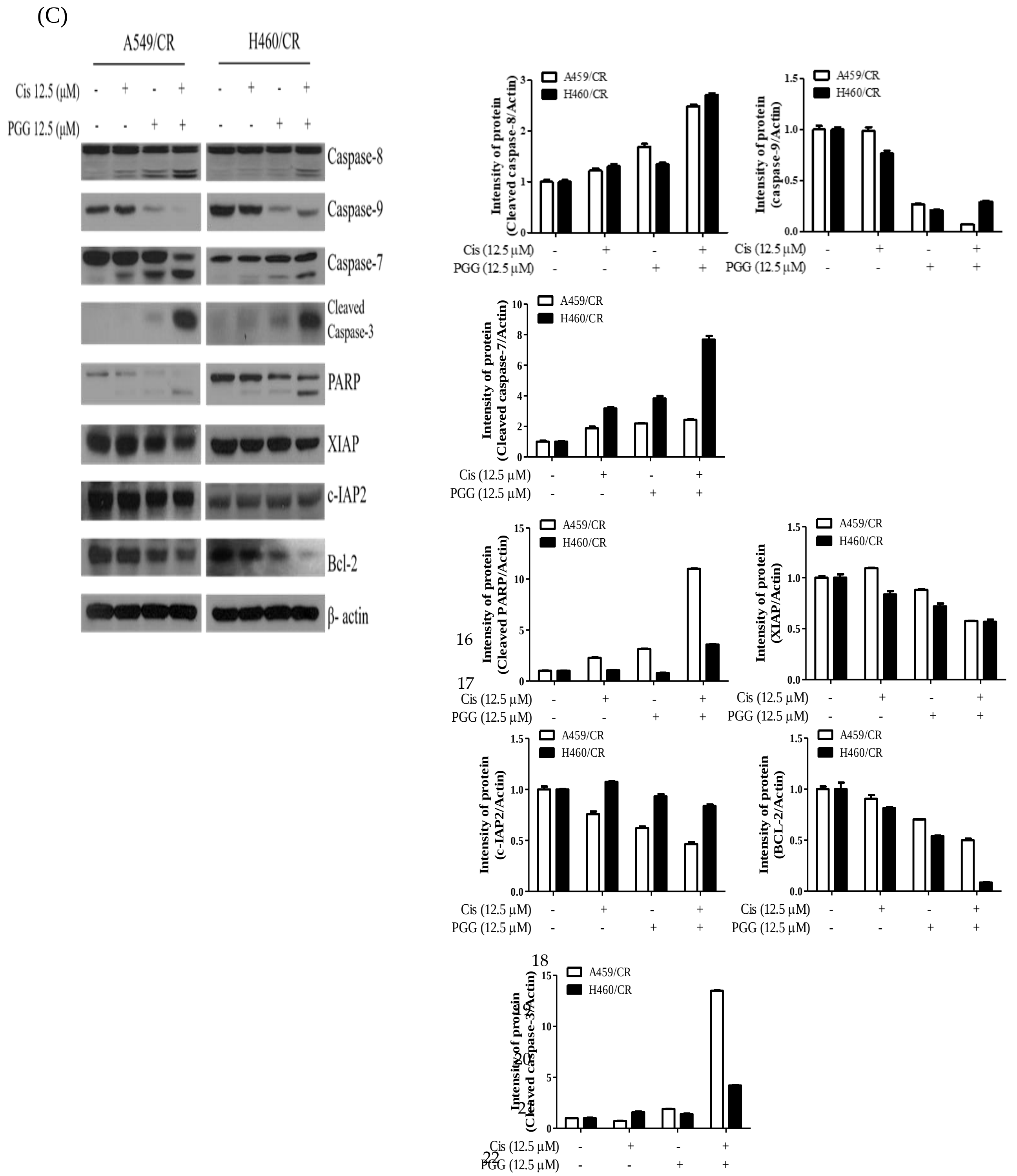

Figure S1. Synergistic effects of PGG and cisplatin on the morphology, viability, sub-G1 accumulation and apoptosis-related proteins in A549/CR and H460/CR cells. (**A**) Cell viability was determined by an MTT assay after A549/CR and H460/CR cells were treated with PGG (12.5 μM) and/or cisplatin (12.5 μM) for 48 h. Then, the synergy between PGG and cisplatin was evaluated by Combination Index (CI) determined by the Chou–Talalay method and CalcuSyn software (Biosoft, Ferguson, MO, USA). CIs below 1 were considered to indicate significant synergy. ##, $p < 0.01$, ###, $p < 0.001$. (**B**) Cell cycle distribution was analyzed by flow cytometry (left) after A549/CR and H460/CR cells were treated with PGG (12.5 μM) and/or cisplatin (12.5 μM) for 48 h. Bar graphs represent the percentages of sub-G1 DNA contents undergoing apoptosis (right). Data represent means ± S.D. *, p < 0.05 vs. cisplatin-treated control. (**C**) A549/CR and H460/CR cells were treated with PGG (12.5 μM) and/or cisplatin (12.5 μM) for 48 h and were subjected to Western blotting for Caspase-8, Caspase-9, Caspase-7, Caspase-3, PRAP, XIAP, c-IAP2, BCL-2 and β-actin.

(A)

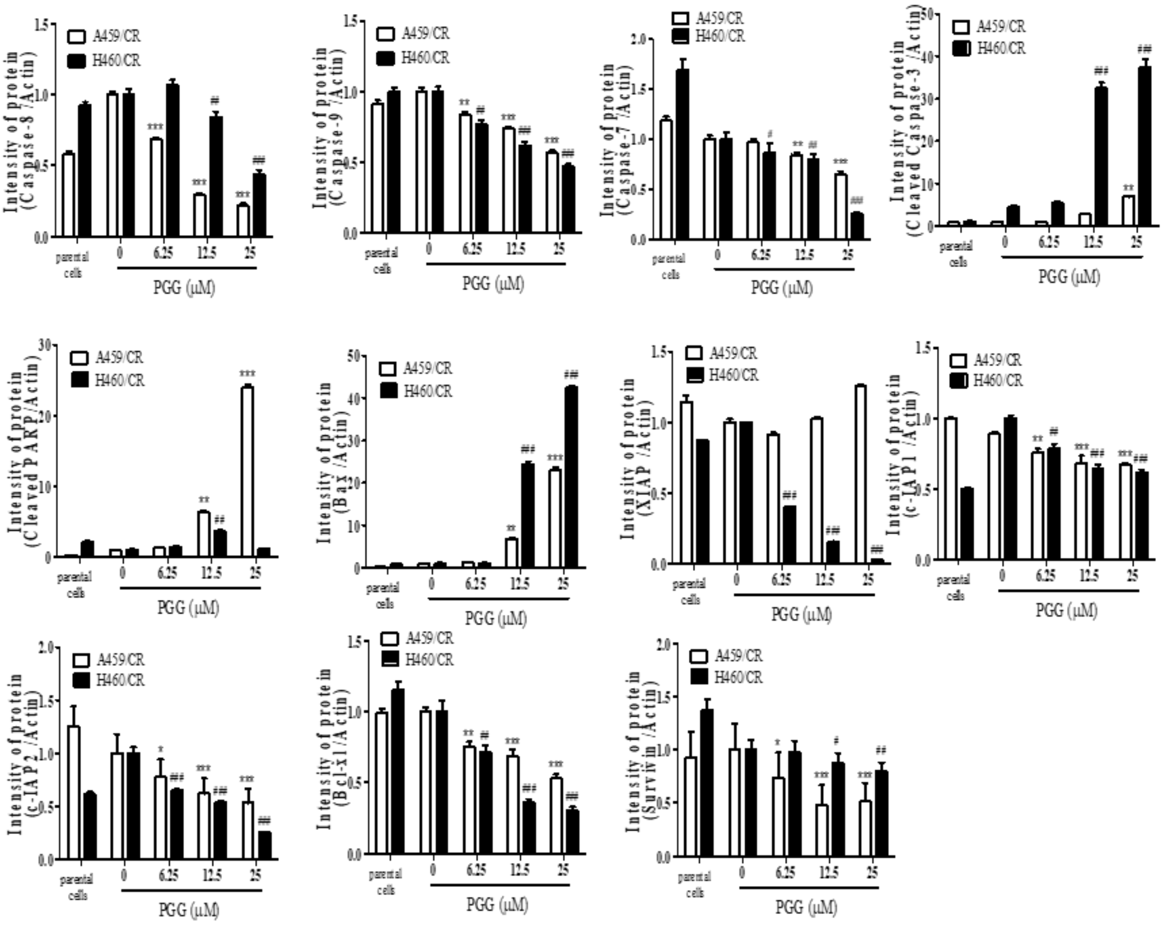
A459/CR
H460/CR
Intensity of protein (Caspase-8 /Actin)
Intensity of protein (Caspase-9 /Actin)
Intensity of protein (Caspase-7 /Actin)
Intensity of protein (Cleaved Caspase-3 /Actin)
Intensity of protein (Cleaved PARP/Actin)
Intensity of protein (Bax /Actin)
Intensity of protein (XIAP /Actin)
Intensity of protein (c-IAP1 /Actin)
Intensity of protein (c-IAP2 /Actin)
Intensity of protein (Bcl-xl/Actin)
Intensity of protein (Survivin /Actin)
parental cells
0
6.25
12.5
25
PGG (μM)

(B)

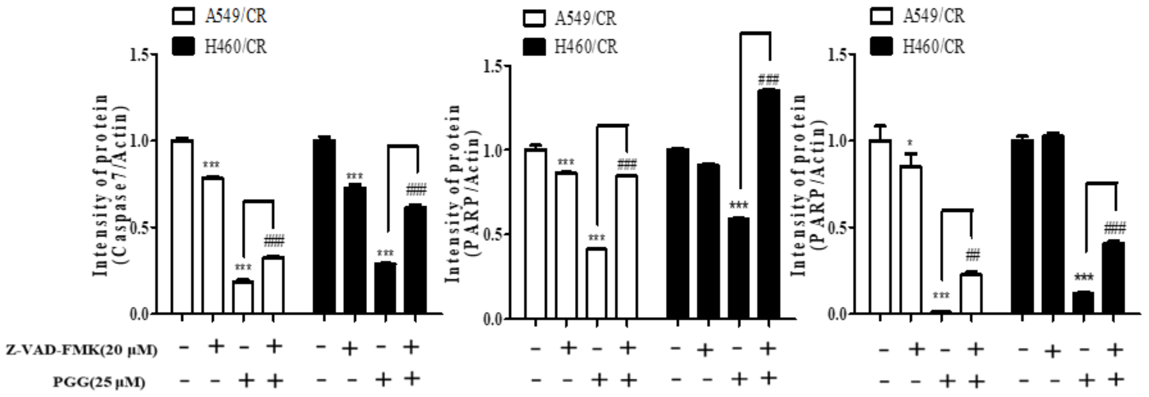
A549/CR
H460/CR
Intensity of protein (Caspase7/Actin)
Intensity of protein (PARP/Actin)
Z-VAD-FMK(20 μM)
PGG(25 μM)

(C)

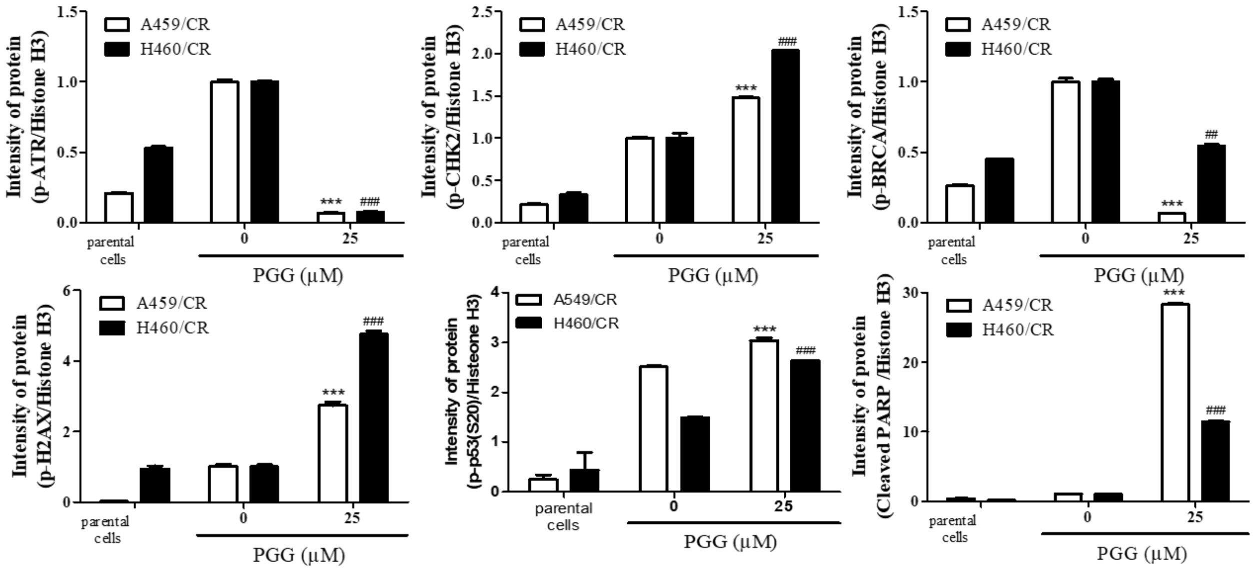
A459/CR
H460/CR
Intensity of protein (p-ATR/Histone H3)
Intensity of protein (p-CHK2/Histone H3)
Intensity of protein (p-BRCA/Histone H3)
Intensity of protein (p-H2AX/Histone H3)
A549/CR
Intensity of protein (p-p53(S20))/Histeone H3)
Intensity of protein (Cleaved PARP /Histone H3)
parental cells
0
25
PGG (μM)
***
###
##
**

(D)

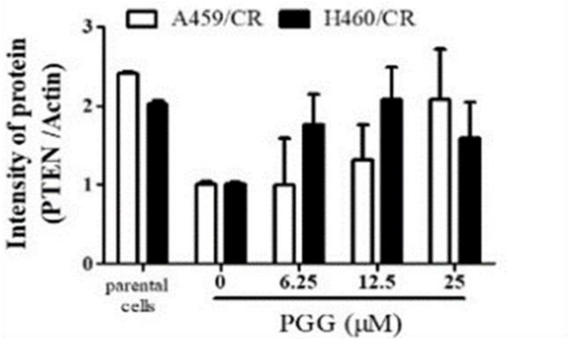
A459/CR
H460/CR
Intensity of protein (PTEN /Actin)
parental cells
0
6.25
12.5
25
PGG (μM)

(E)

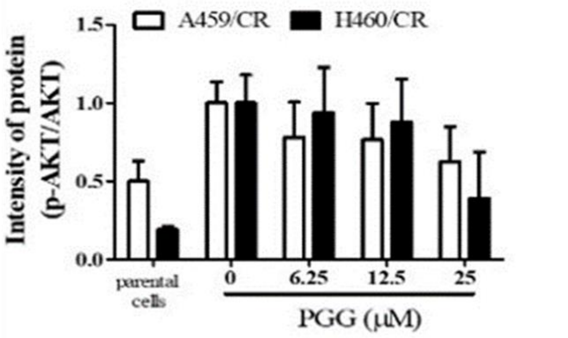
A459/CR
H460/CR
Intensity of protein (p-AKT/AKT)
parental cells
0
6.25
12.5
25
PGG (μM)

(F)

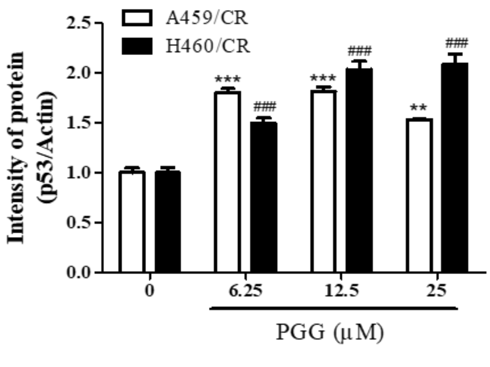
A459/CR
H460/CR
Intensity of protein (p53/Actin)
0
6.25
12.5
25
PGG (μM)
***
**
###

(G)

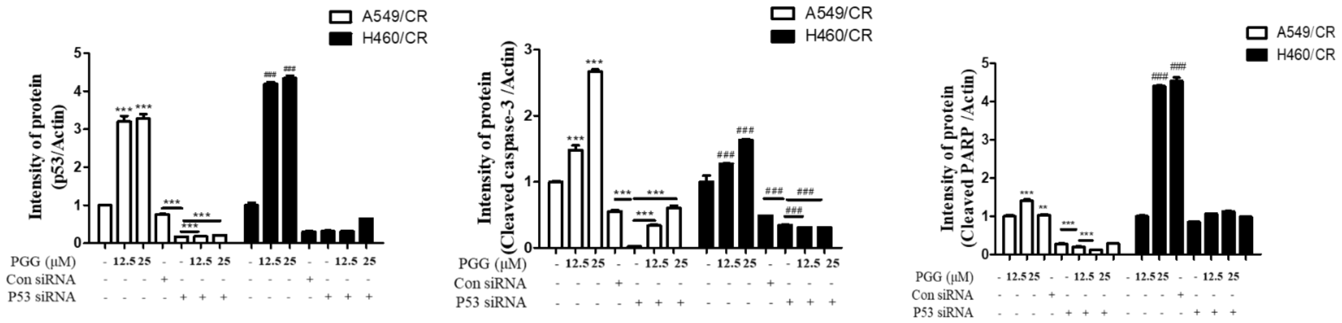

Figure S2. Quantification of Western blotting. Bar graphs for (**A**) Figure 3B, (**B**) Figure 3C, (**C**) Figure 4C, (**D**) Figure 5A, (**E**) Figure 5B, (**F**) Figure 6A and (**G**) Figure 6D. Data represent means ± SD. *, $p < 0.05$, **, $p < 0.01$,***p < 0.001, #, $p < 0.05$, ##, $p < 0.01$, ###p < 0.001 vs. untreated control.
